# Vibronic Coupling in Spherically Encapsulated, Diatomic Molecules: Prediction of a Renner–Teller-like Effect for Endofullerenes

**DOI:** 10.1021/acs.jpca.1c10970

**Published:** 2022-03-08

**Authors:** Andreas W. Hauser, Johann V. Pototschnig

**Affiliations:** Institute of Experimental Physics, Graz University of Technology, Petersgasse 16, 8010 Graz, Austria

## Abstract

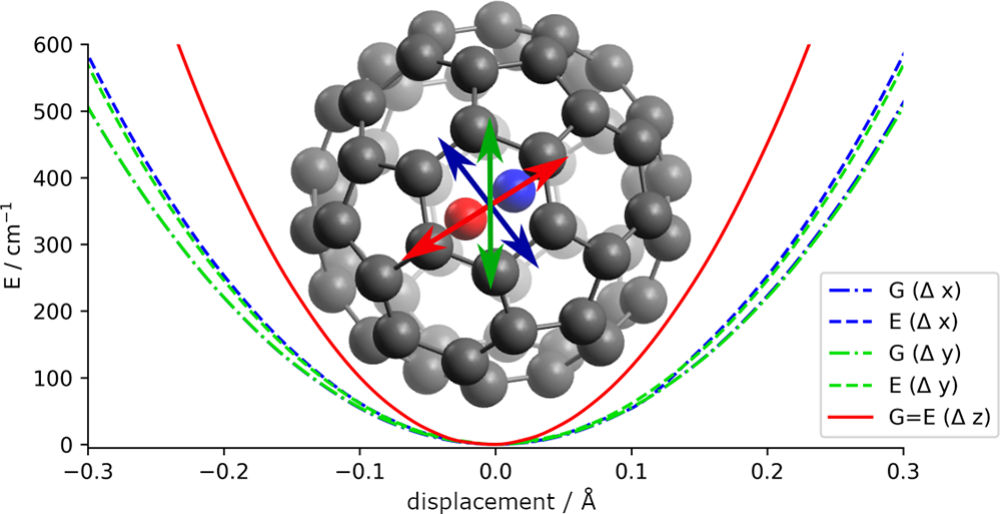

In the year 1933, Herzberg and Teller realized that the potential energy surface of a triatomic, linear molecule splits into two as soon as the molecule is bent. The phenomenon, later dubbed the Renner–Teller effect due to the detailed follow-up work of Renner on the subject, describes the coupling of a symmetry-reducing molecular vibration with degenerate electronic states. In this article, we show that a very similar type of nonadiabatic coupling can occur for certain translational degrees of freedom of diatomic, electronically degenerate molecules when trapped in a nearly spherical or cylindrical quantum confinement, e.g., realized through electromagnetic fields or molecular encapsulation. We illustrate this on the example of fullerene-encapsulated nitric oxide, and provide a prediction of its interesting, perturbed vibronic spectrum.

## Introduction

In its simplest form, the Renner–Teller (RT) effect, as first described by Herzberg and Teller,^1^ and a year later thoroughly discussed by Renner,^2^ occurs in linear, open-shell molecules consisting of three atoms. In these systems, the bending vibration, which breaks the rotational symmetry present along the internuclear axis, couples to the electronic motion and enforces a local breakdown of the Born–Oppenheimer approximation. Although this perturbation does not lift the degeneracy of the coupled electronic states in first order, it has significant, well-studied consequences for the vibronic as well as the ro-vibronic spectrum of these molecules; the latter is due to the fact that the corresponding eigenfunctions of the molecular Hamiltonian are no longer eigenfunctions of the electronic orbital angular momentum as well as the vibrational angular momentum operator.^3^ A recent overview of the subject has been provided by Jungen.^4^ Initiated by the early work of Pople,^5^ interest in spin–orbit coupling effects on the vibronic spectra of linear molecules emerged. For relativistic treatments, see refs (6−8).

**Figure 1:**
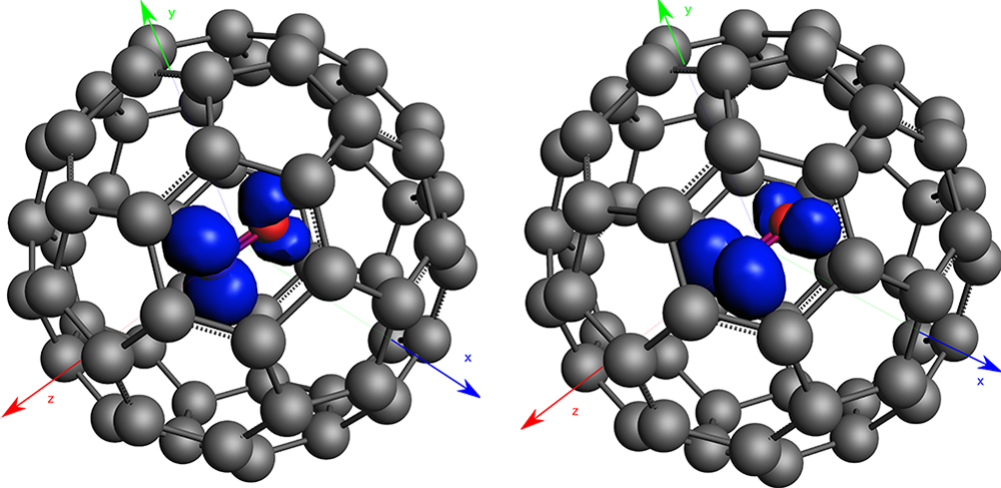
Spin isodensity plots (blue) of the two degenerate electronic states of NO@C_60_. With NO aligned with the *C*_3_ axis, this endofullerene is a representative of the *C*_3*v*_ molecular point group.

In this article, we show that a similar type of nonadiabatic coupling must occur in the vibronic spectra of electronically degenerate, spherically encapsulated diatomic molecules, a molecular arrangement most closely realized by endofullerenes, i.e., small molecules enclosed by fully intact fullerene cages. We propose that, in such a setup, with cage dimensions similar to the de Broglie wavelength of the trapped molecule, the necessarily quantized translational motions are able to lift an electronic degeneracy in second order. These interesting, yet somewhat exotic molecular systems can be synthesized via “molecular surgery”,^9−13^ a process comprising the chemical opening of the cage, embedding of a guest molecule, and a follow-up reconstruction of the carbon confinement. Degenerate electronic states, a necessity for the RT effect, are mostly found in electronically excited states, molecular radicals, or molecular ions, which poses an experimental difficulty. We have picked the NO molecule for the sake of a preliminary, yet meaningful first investigation of the proposed nonadiabatic effect. This small diatomic molecule features a ^2^Π degenerate ground state, which makes it a computationally feasible, but also experimentally accessible test object for follow-up spectroscopic investigations.^14−16^ Density functional theory (DFT) will be employed to obtain the potential energy surfaces (PES). Alternatively, diatomic molecules such CCl and NS, which are also characterized by a ^2^Π degenerate electronic ground state, should show a similar Renner–Teller-like effect in the far- to mid-infrared spectrosopic region. Different positions and alignments are possible for such an encapsulated diatomic molecule.^17−20^ In the case of NO, a structural optimization without any symmetry enforcement shows that the minimum energy is reached if the NO is in the center of the C_60_ cage, and the N–O axis is almost in line with the *C*_3_ rotational axis of the fullerene connecting the centers of two opposite hexagons. The two ^2^Π states become exactly degenerate if a perfect alignment between the internuclear axis and the *C*_3_ rotational axis is enforced. In this slightly idealized geometric arrangement, the molecular system, as shown in Figure 1, is a representative of the *C*_3*v*_ molecular point group.

Neglecting for a moment the vibrational degrees of freedom of the fullerene itself and looking at a single, encapsulated diatomic molecule perfectly aligned to the *C*_3_ axis of the fullerene, it is obvious that the remaining vibrational degree of freedom of this diatomic molecule can not cause the proposed coupling; neither can a translation along the *C*_3_ axis. Let us refer to the latter as the *z*-axis in the laboratory frame. However, the two remaining translational motions, i.e., motions displacing the diatomic along the *x* and *y*-axis, are now taking the role of the 2-fold degenerate bending vibrations as it would occur in the case of a Renner–Teller-active molecule. Another way of thinking about this type of coupling is to interpret the center-of-mass of the fullerene cage as the missing “third” atom. Obviously, the fullerene cage is not perfectly spherical as it consists of distinct atoms, a fact that will show up below when discussing one-dimensional cuts through the actual PES obtained from electronic structure theory. However, the deviation from the typical PES of Renner–Teller effect theory is negligible when discussing vibronic features exclusively. Experimentally, diatomics encapsulated in fullerenes have been investigated by UV to far-infrared spectroscopy,^21−23^ inelastic neutron scattering,^24−26^ nuclear magnetic resonance,^27^ and photoelectron spectroscopy.^28^ Infrared spectroscopy of molecules, which allows to study low lying rovibronic states showing the proposed effect, either requires a permanent electric dipole moment or induction effects, which can appear in C_60_ due to interactions of the guest molecule with its confinement.^21,22^ On the theoretical side, the enclosure of heteronuclear diatomics or triatomics has been studied for the case of CO@C_60_^29^ (bond distances and vibrational frequencies) and H_2_O@C_60_.^30^ Regarding open shell systems, a comparison between singlet and triplet spin manifolds of encapsulated B_2_, O_2_ and Ge_2_ can be found in ref (31). For HF@C_60_, bond elongation and a quenching of the vibrational frequency have been predicted.^32^ Besides the electronic and vibrational states studied here, rotational coupling can lead to additional splittings; see, e.g., refs (33−39) for details.

## Theory

The ^2^Π ground state of the NO molecule shows a 2-fold degeneracy with respect to Λ = ±1, the projection of the orbital angular momentum onto the internuclear axis. Strictly speaking, these quantum numbers are valid only for the free linear molecule. Fortunately, as will be shown below, symmetry deviations introduced by the fullerene cage are minimal. The sign of Λ dictates the behavior of the corresponding electronic wave function with respect to a rotation *R̂* around the *z*-axis by an arbitrary angle ϕ,
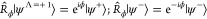
1In the case of the “Renner–Teller-like” nonadiabatic coupling proposed in this work, the RT-active doubly degenerate bending mode is replaced by the translational motions of the NO molecule perpendicular to the internuclear axis. Identifying the latter with the *z*-axis of the laboratory frame (and also the *C*_3_ axis of the fullerene), we can identify *q*_*x*_ and *q*_*y*_, the translations in the *x* and the *y* directions, as suitable replacements. In analogy to the handling of the electronic part described above, we introduce complex nuclear coordinates
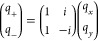
2as a basis in which the operator *C*_ϕ_ for an arbitrary rotation of the angle ϕ becomes diagonal, with eigenvalues e^*iϕ*^ and e^–*iϕ*^, respectively. With this choice, the usual Renner–Teller Hamiltonian can be employed. Written in polar coordinates for the two Λ values it has the form^40^
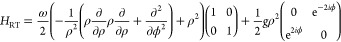
3A diagonalization of the potential part of the Hamiltonian yields two adiabatic electronic PES, again formulated in polar coordinates:
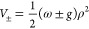
4The first part of the Hamiltonian corresponds to the isotropic two-dimensional Hamiltonian with the solutions^33,41^
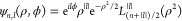
5expressed via the associated Laguerre polynomials *L* with eigenvalues of *E*_*n*,*l*_ = ν̅(*n* + 1). For the evaluation of the distance-dependent dipole moments we introduce the length unit^41^, with μ denoting the reduced mass. In order to obtain the corresponding vibronic eigenstates of *H*_RT_, the coupling terms (second term on the rhs of eq 3) are added and the obtained matrix is diagonalized. This procedure requires the evaluation of matrix elements ⟨*n*,*l*|*gρ*^2^e^–2*iϕ*^|*n*,*l*⟩, which are known analytically^41^ and are evaluated via SymPy, a Python library for symbolic computation. The total angular momentum **J** of a given vibronic state with quantum number *j* is obtained by coupling the vibrational angular momentum **K** (eigenfunctions |ψ_*n*,*l*_⟩) and the orbital angular momentum **L** (eigenfunctions |ψ^+^⟩, |ψ^–^⟩). Coupled states are denoted as |Φ_*j*,ν_⟩. Spectral intensities are proportional to the absolute square of the dipole transition moment,
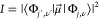
6Introducing the expectation values of the dipole moment operator in the laboratory frame,
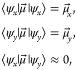
7which can be extracted from the DFT calculations as a function of *r* (see the Supporting Information for details), spectral intensities can be obtained by taking the absolute square of the bracketed expressions
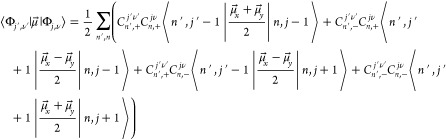
8Note the expansion of the vibrational part in a basis of eigenfunctions for the isotropic 2D harmonic oscillator according to eq 5.

## Computational Details

Regarding the actual PES calculation, we place the NO molecule in a C_60_ cage and optimize the geometry at the DFT level of theory, using the TZP basis set available in ADF as part of the SCM software package.^42^ The PBE functional^43^ is employed together with a D3 dispersion correction.^44^ DFT output files and code are provided in a repository.^45^ An interaction energy
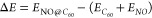
9of −0.165 eV is obtained if *C*_3*v*_ symmetry is enforced. The corresponding molecular geometry, as depicted in Figure 1, features an exact electronic degeneracy due to a perfect alignment with the *C*_3_ axis of the fullerene, but breaks icosahedral symmetry. In a DFT-based frequency computation within the *C*_3*v*_ symmetry the three lowest modes correspond to a translational movement of NO within the C_60_ cage. The two degenerate modes, perpendicular to the internuclear axis, have a frequency of 122.5 cm^–1^. In our model Hamiltonian, these modes will be treated like RT-active bending modes. The translational in-axis movement of the NO molecule is found at 165.7 cm^–1^. Within RT effect theory, the latter can be related to the inactive asymmetric stretching mode, which has about the same intensity in the DFT computation as the degenerate modes. Vibrational modes involving the carbon cage start at 259.7 cm^–1^, but are of negligible intensity. The lowest vibrational transitions of C_60_ with significant intensity (about a factor of 1300/1000/200 stronger than the translational modes) can be found at 522.6/581.8/1180.7 cm^–1^, which agrees very well with the experimentally confirmed lowest lines of C_60_ at 526/576/1183 cm^–1^, respectively.^46^ The N–O bond-stretching vibration can be found at 1894.9 cm^–1^ (with an equilibrium bond distance of 1.163 Å) and is about a factor of 140 stronger in intensity than the translational modes. Note the slight difference in comparison to the vibration of the free NO molecule in gas phase: Applying the same computational method, we obtain a bond distance of 1.167 Å and a frequency of 1866.1 cm^–1^ for free NO, which indicates a minimal compression of the molecule inside the C_60_ cage.^47^ Experimentally, the lowest vibrational level of gas phase NO is found at 1876 cm^–1^,^16^ which validates the accuracy of our computational treatment. The confinement has a minimal impact on the rotational constant of NO, rising it by less than one percent to a computed value of 1.67 cm^–1^. Similarly, a negligible mode coupling between NO translations and low lying vibrations of the C_60_ cage can be expected from a comparison of fullerene and endofullerene frequencies (see Supporting Information for details).

As can be seen in Figure 1, the spin density, i.e., the electron density of the highest singly occupied molecular orbital (HOMO), is clearly dominated by the p-orbitals of the oxygen and nitrogen atoms. At *C*_3*v*_ symmetry, the p_*x*_ and the p_*y*_ orbitals are degenerate, giving rise to the 2-fold electronic degeneracy needed for the RT interaction. Displacement orthogonal to the *C*_3_ axis of the NO molecule reduces the symmetry of the system. We choose the *x*-axis of our coordinate system in such a way that the *xz*-plane is cutting the hexagons on the top and the bottom of the fullerene exactly at the middle of a C–C bond. In this case, a translation of the NO molecule along the *x*-axis reduces the overall symmetry to C_*s*_, as it leaves a single mirror plane (*xz*) as symmetry element. A translation along the *y*-axis, however, breaks any symmetry and reduces the system to *C*_1_. This introduces a certain complication in the calculations since the two coupled electronic states can no longer be distinguished by symmetry, which enforces the application of an excited state method. We employ time-dependent DFT for their computation. The inevitable difference in absolute energies with respect to ordinary DFT is compensated by an *ad hoc* shift of 0.28 eV. Corrections of this magnitude with respect to absolute energy are expected for this method;^48,49^ the accuracy of relative energies, i.e., the shape of the PES, has been confirmed through a direct comparison to ordinary DFT results where possible, with deviations in the range of a few wavenumbers (see Supporting Information for details).

## Results and Discussion

**Figure 2:**
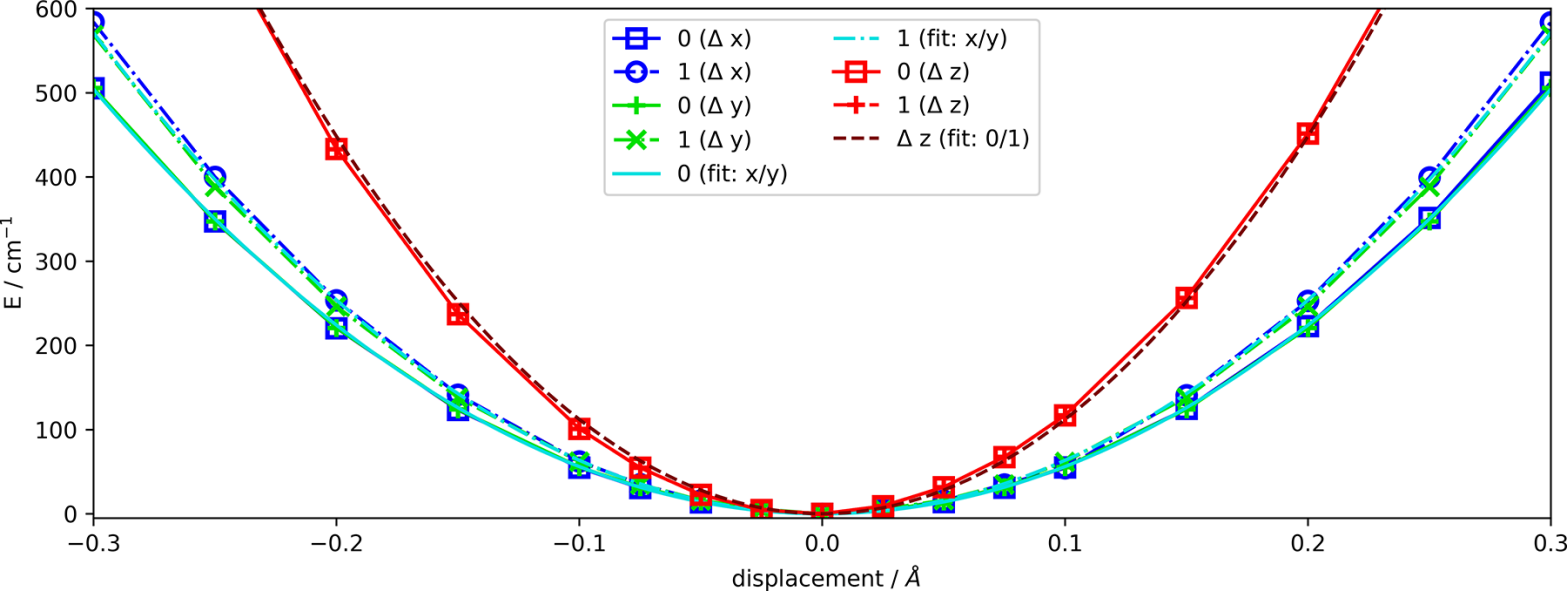
Potential energy curves for the two coupled electronic states (denoted as 0 and 1) while displacing the NO molecule within C_60_ (Δ*x*, Δ*y*, Δ*z*). Displacements Δ*x* and Δ*y* produce the two curves of eq 4.

**Figure 3:**
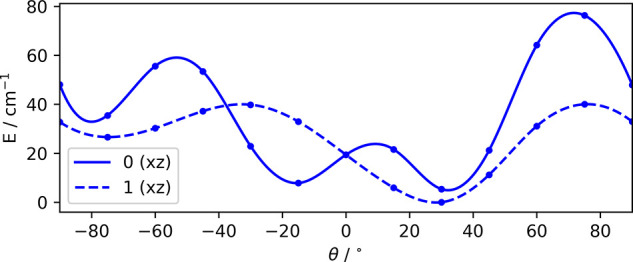
Energy dependence of the two involved electronic states for a rotation of the NO molecule within the C_60_ cage. For symmetry reasons, the warping of both PES repeats itself between 90 and 270°.

An overview of the PES cuts obtained by displacements of the NO molecule is given in Figure 2. In these computations the C_60_ was kept fixed and the NO molecule was displaced by *Δx*, *Δy*, and *Δz*. We use different colors for the three displacements in space (blue, green, and red, respectively). The two electronic states are distinguished by solid and dashed line styles. Note that, for symmetry reasons, deviations between the scans along *x* and *y* directions, as well as along the positive and negative axis, are a direct measure of the PES warping due to the icosahedral cage structure. However, the minimal variation documented in Figure 2 justifies the simplification of assuming cylindrical symmetry. Therefore, *x* and *y* branches of the same state have been fitted together. Displacements in *Δz* (red), i.e. the nonactive mode, show a larger curvature and are not fully symmetric due to the heterogeneous nature of the diatomic molecule. This type of displacement does not lift the degeneracy of the two electronic states (numerical deviations are below 1 cm^–1^). For the sake of completeness we have also investigated rotations of NO inside the fullerene. Energies as a function of the rotational angle for motions in the *xz* plane are given in Figure 3 for the two different electronic states. A barrier height of about 75 cm^–1^ has to be overcome for unhindered rotations, which corresponds to a temperature of about 110 K. Interestingly, the two electronic states show a different angular dependence, which opens a new chapter for future investigation, as it suggests a nonadiabatic coupling also on the level of rotational excitation. It is also apparent that the high-symmetry orientation along the *C*_3*v*_ axis (θ = 0°) is not exactly the global minimum. Note that, within a Cartesian coordinate picture, a rotation of the caged molecule, e.g., within the *xz*-plane means that the rotational axis agrees with the symmetry axis of the p_*y*_-lobe. Therefore, the only modification of this orbital, and therefore its corresponding state energy, with the angle θ, is due to its electronic coupling to the p_*x*_-state, and not directly through the confining cage. The same holds for the p_*x*_-component in case of a rotation within the *yz*-plane.

Recently, a review article has been dedicated to the topic of translation-rotation dynamics and spectroscopy of light-molecule endofullerenes.^36^ On the theory side, powerful methods such as the discrete variable representation^50,51^ exist for the numerical solution of low-dimensional quantum motion, but will have to be extended to cover nonadiabatic coupling effect at hand. Particularly useful will be the ansatz of Kalugina and Roy,^52^ who expanded the PES of HF in C_60_ in a basis of bipolar spherical harmonics. Regarding future experimental work, this system might offer the possibility to investigate nonadiabatic coupling for hindered rotations via microwave spectroscopy.^23^

**Figure 4:**
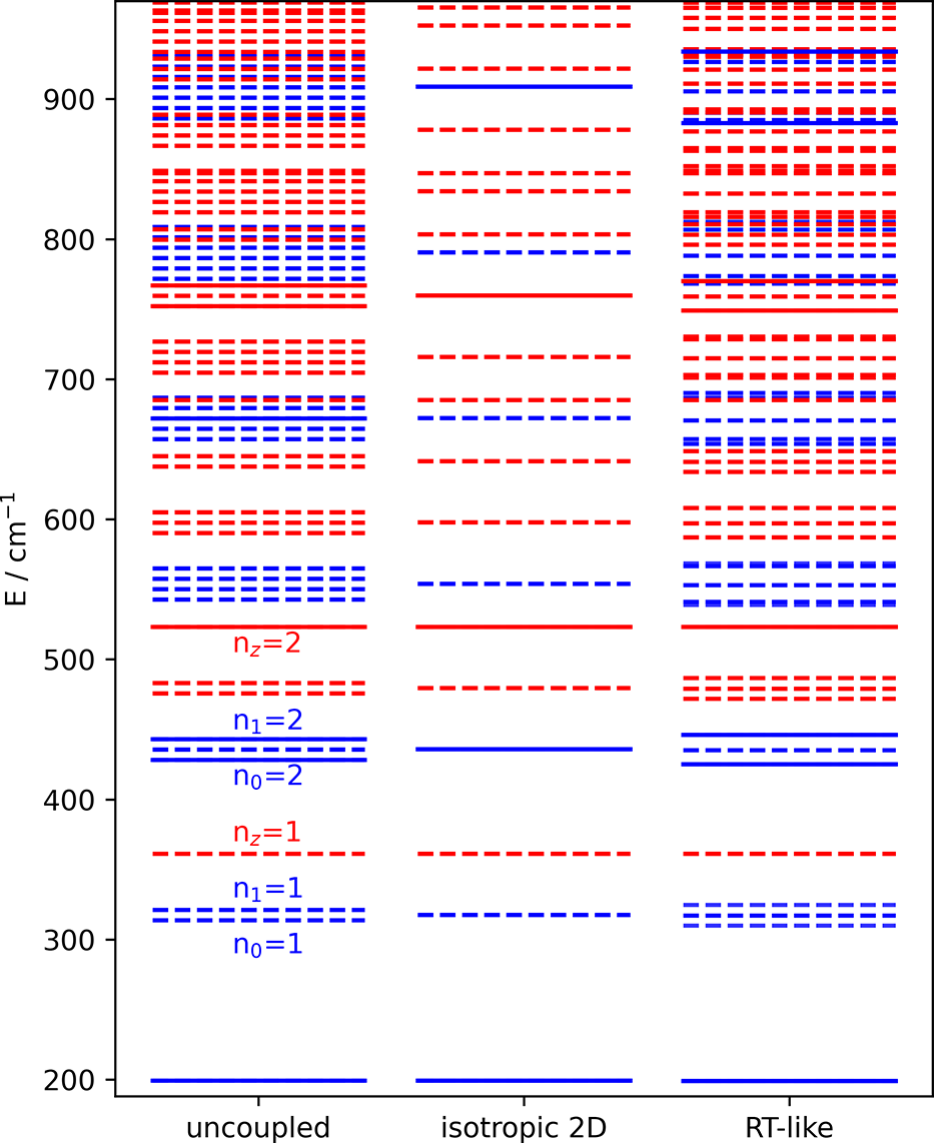
Energy levels for two uncoupled harmonic oscillators (left), for an isotropic 2D harmonic oscillator (middle), and obtained by diagonalization of the RT-like Hamiltonian (right). Solid/dashed lines indicate levels with significant/negligible transition probability from the ground state at 200 cm^–1^. Levels with *n*_*z*_ = 0 are printed in blue; levels with *n*_*z*_ > 0 are printed in red.

In the next step, fitting parameters are extracted from the PES scans in *Δx* and *Δy*, and used to set up the vibronic Hamiltonian for a treatment within RT effect theory. We obtain ω = 118.3 cm^–1^ and *g* = 7.4 cm^–1^ for the parameters in eq 3. Matrix diagonalization produces the energy levels shown in the right panel of Figure 4. In order to illustrate the impact of the nonadiabatic coupling, we compare them to the level structure of two uncoupled one-dimensional harmonic oscillators (left, ω_0_ and ω_1_) and a two-dimensional harmonic oscillator (middle, using the average ω = (ω_0_ + ω_1_) /2). Levels indicated by solid lines have a nonzero transition probability and are accessible from the ground state (lowest blue line at 200 cm^–1^).

**Figure 5:**
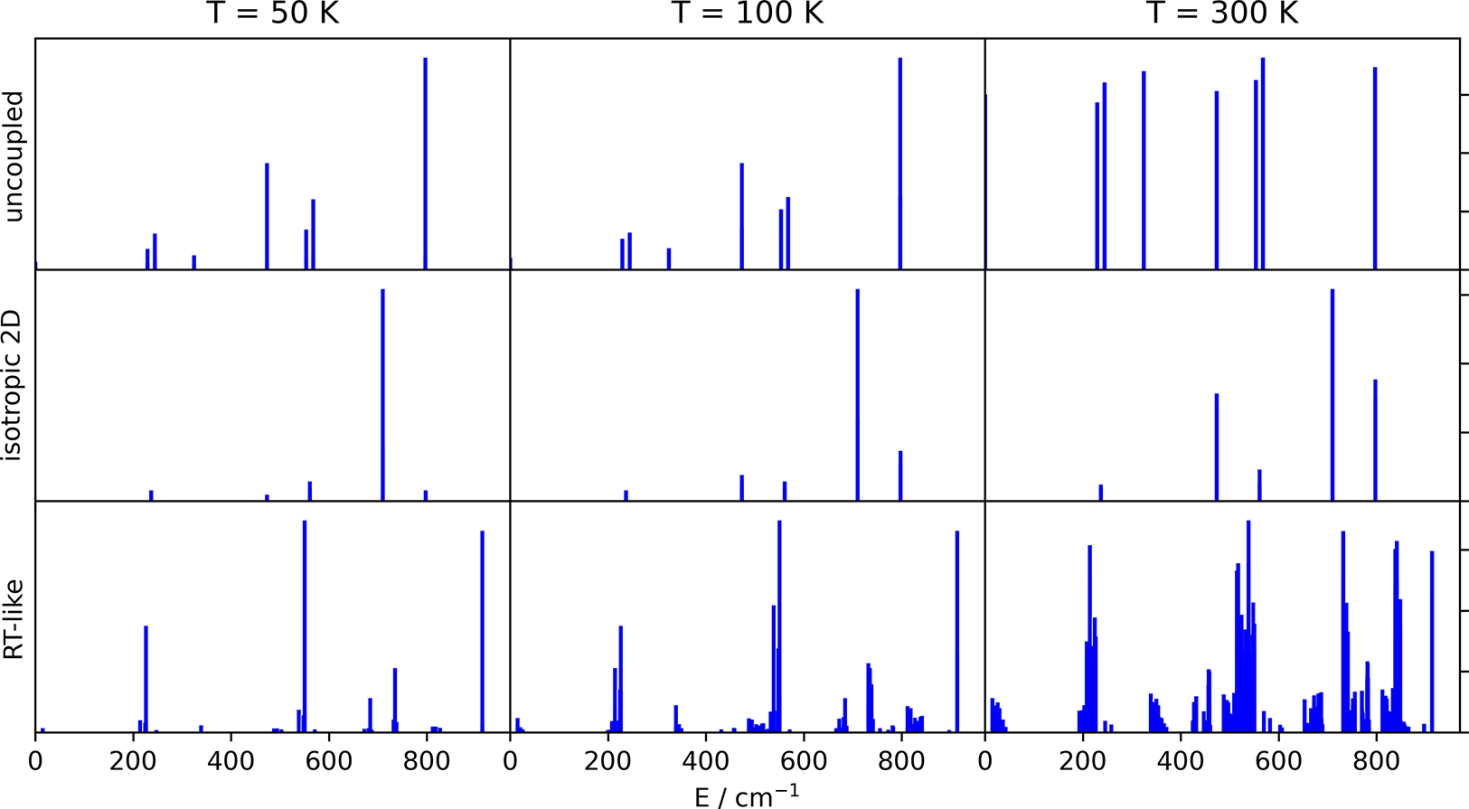
Infrared spectra derived from the energy level structures obtained for two uncoupled harmonic oscillators and an isotropic 2D harmonic oscillator and were obtained by diagonalizing the Hamiltonian described in the Theory.

Note the pronounced deviations from an equidistant spacing due to the nonadiabatic interaction, but also the rather small splitting in comparison to level splittings observed for actual Renner–Teller active modes^4^ involving vibrational instead of translational degrees of freedom. As is apparent from Figure 4 transitions with *Δn* = ± 1 have no intensity, while *Δn* = ±2 transitions are strong, which can be explained by the dipole moment dependence. For small displacements, it is dominated by the permanent electric dipole moment of the nitric oxide, which shows only a minimal variation in the *x*- and *y*-direction, and is therefore favoring *Δn* = ±2 transitions. Together with information on line intensity derived from eq 8, we can make predictions for the infrared absorption spectra at various temperatures. Our results are shown in Figure 5 for 50, 150, and 300 K. Even at temperatures as low as 50 K, which are accessible through cooling,^23^ this RT-like coupling leads to significant deviations from the expected vibronic spectrum of a spatially confined diatomic molecule. Hot band excitations with *n*_*z*_ = 1, i.e., the first excited state in the nonactive mode (165.7 cm^–1^), are already contributing at lower temperatures and give a significant contribution to the spectra at room temperature. Numerical values of intensities and line positions are provided in the Supporting Information.

## Conclusion

In conclusion, we are predicting a new type of nonadiabatic coupling for electronically degenerate diatomic molecules in spherical confinements. Technically, it can be handled similar to the standard procedure in Renner–Teller effect theory if the translations perpendicular to the internuclear axis are treated like an RT-active mode. Given the example of NO encapsulated in C_60_, we have calculated vibronic levels and intensities, and demonstrated that this coupling should lead to significant, experimentally accessible deviations in the infrared absorption spectrum for this class of molecular systems. Similar effects are to be expected for electronically degenerate diatomics in any type of spherical or cylindrical confinement, e.g. in ion traps, magneto-optical traps, carbon nanotubes, or metal–organic frameworks of suitable molecular structure.

## Supporting Information Available

Dipole moment analysis as a function of the NO displacement, DFT and TD-DFT potential energy surface comparison, tables of spectral lines and intensities at various temperatures, and vibrational modes of C_60_ and NO@C_60_ (PDF)
