# Supplementary material for: Vibronic Coupling in Spherically Encapsulated, Diatomic Molecules: Prediction of a Renner–Teller-like Effect for Endofullerenes
Source: J Phys Chem A. 2022 Mar 8;126(10):1674–80. doi: 10.1021/acs.jpca.1c10970 (PMC8935370; doi:10.1021/acs.jpca.1c10970)
Supplement: Supplementary file 1 — jp1c10970_si_001.pdf [file jp1c10970_si_001.pdf]

Supporting Information:
Vibronic Coupling in Spherically Encapsulated, Diatomic Molecules: Prediction of a Renner-Teller-like Effect for Endofullerenes

Andreas W. Hauser*[1] and Johann V. Pototschnig[1]

[1]*Institute of Experimental Physics, Graz University of Technology, Petersgasse 16, A-8010 Graz, Austria*

(Dated: February 16, 2022)

Keywords: Renner-Teller effect, molecular spectroscopy, vibronic coupling, Born-Oppenheimer, non-adiabatic

In Section 1 of this Supporting Information the dipole moment is analyzed as a function of the NO displacement. All expectation values needed in the main manuscript are based on integrals over these functions. Section 2 contains tabulated information on the actual lines and intensities obtained by the application of Renner-Teller effect theory to the non-adiabatic coupling between two translational modes of NO and its two-fold degenerate electronic ground state. In Section 3 we compare the results of standard DFT and TD-DFT in a PES scan where both methods can be employed. Section 4 lists the vibrational modes of C_{60} and NO@C_{60} for a direct comparison.

I. PERMANENT ELECTRIC DIPOLE MOMENTS

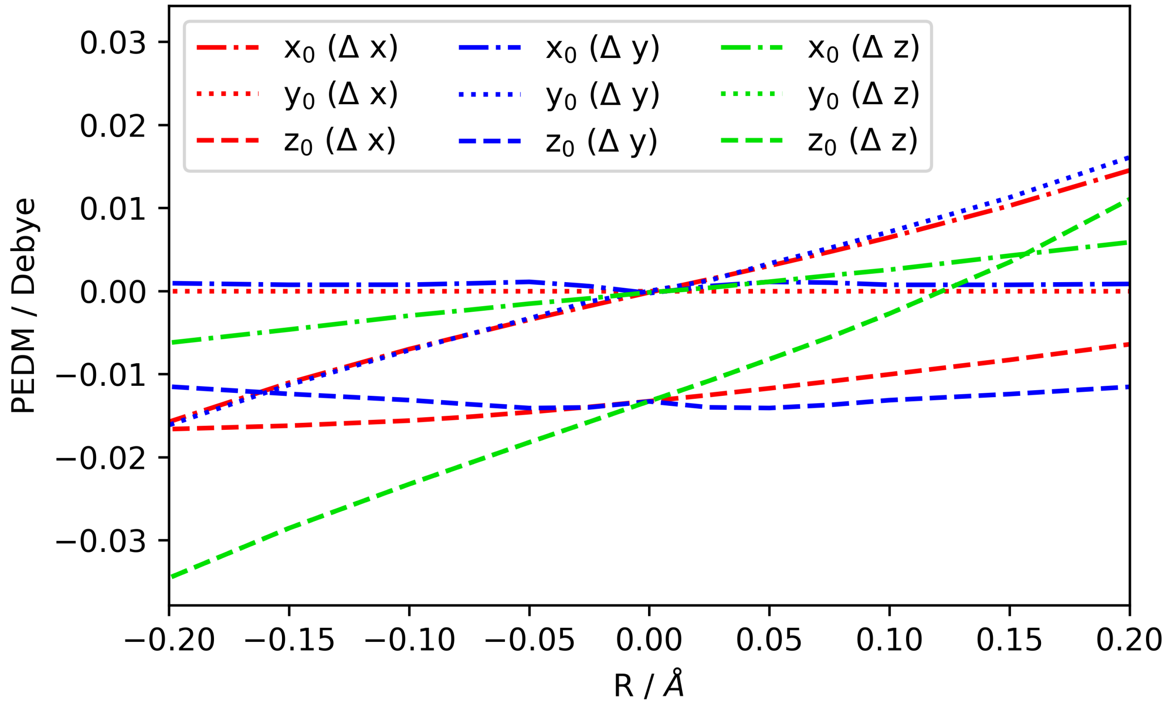

FIG. S1: Permanent electric dipole moment of NO@C_{60}. The different components of of the dipole moment vector (x,y,z) are shown as a function of x-, y- and z-displacements within the fullerene cage.

* Author to whom correspondence should be addressed. Electronic mail: andreas.w.hauser@gmail.com

II. TABULATED SPECTRA

TABLE I: The transition energy (T_e) in cm^{-1} and intensity in arbitrary units for the spectra shown in the main text for a Temperature of 50 K. Only lines of non-negligible intensity (above 2e-5) are presented in the table.

T_e	I	n_z lower	n_z upper	T_e	I	n_z lower	n_z upper
214.267	2.887137e-05	0	0	223.869	2.110068e-05	0	0
226.042	2.924775e-05	0	0	226.042	2.914412e-04	0	0
538.241	5.791057e-05	0	2	547.843	4.232402e-05	0	2
550.016	5.866554e-05	0	2	550.016	5.845767e-04	0	2
683.761	9.068920e-05	0	0	731.462	3.108672e-05	0	0
734.941	1.737628e-04	0	0	738.127	2.387009e-05	0	0
912.752	5.553527e-04	0	0	912.752	2.082184e-05	1	1
912.752	2.082184e-05	1	1				

TABLE II: The transition energy (T_e) in cm^{-1} and intensity in arbitrary units for the spectra shown in the main text for a Temperature of 100 K. Only lines of non-negligible intensity (above 2e-5) are presented in the table.

T_e	I	n_z lower	n_z upper	T_e	I	n_z lower	n_z upper
14.776	2.071061e-05	0	0	214.267	3.885885e-05	1	1
214.267	3.885885e-05	1	1	214.267	1.024563e-04	0	0
223.869	2.547116e-05	1	1	223.869	2.547116e-05	1	1
223.869	6.715797e-05	0	0	226.042	1.717880e-04	0	0
226.042	6.515444e-05	1	1	226.042	6.515444e-05	1	1
338.750	4.154162e-05	0	2	487.639	2.003342e-05	0	0
531.863	3.121864e-05	0	2	538.241	2.055083e-04	0	2
538.241	3.518756e-05	1	3	538.241	3.518756e-05	1	3
546.154	3.285733e-05	0	2	547.843	1.347064e-04	0	2
547.843	2.306470e-05	1	3	547.843	2.306470e-05	1	3
550.016	3.445747e-04	0	2	550.016	3.458000e-05	0	2
550.016	5.899880e-05	1	3	550.016	5.899880e-05	1	3
680.928	2.202863e-05	0	0	683.761	5.345613e-05	0	0
683.761	2.027443e-05	1	1	683.761	2.027443e-05	1	1
731.462	4.184057e-05	1	1	731.462	1.103180e-04	0	0
731.462	4.184057e-05	1	1	734.941	1.024233e-04	0	0
734.941	3.884632e-05	1	1	734.941	3.884632e-05	1	1
738.127	2.881419e-05	1	1	738.127	7.597230e-05	0	0
738.127	2.881419e-05	1	1	811.613	4.018330e-05	0	2
817.954	3.658265e-05	0	2	826.389	2.120740e-05	0	2
838.232	2.274853e-05	0	2	841.169	2.421250e-05	0	2
912.752	2.134762e-05	2	2	912.752	3.273489e-04	0	0
912.752	2.134762e-05	2	2	912.752	1.241544e-04	1	1
912.752	1.241544e-04	1	1	912.752	2.134762e-05	2	2

TABLE III: The transition energy (T_e) in cm^{-1} and intensity in arbitrary units for the spectra shown in the main text for a Temperature of 300 K. Only lines of non-negligible intensity (above 2e-5) are presented in the table.

T_e	I	n_z lower	n_z upper	T_e	I	n_z lower	n_z upper
207.889	2.926542e-05	1	1	207.889	2.344985e-05	2	2
207.889	2.926542e-05	1	1	207.889	2.344985e-05	2	2
207.889	2.344985e-05	2	2	214.267	4.876934e-05	2	2
214.267	3.201705e-05	3	3	214.267	6.086416e-05	1	1
214.267	3.201705e-05	3	3	214.267	4.876934e-05	2	2
214.267	6.086416e-05	1	1	214.267	3.201705e-05	3	3
214.267	3.443601e-05	0	0	214.267	3.201705e-05	3	3
214.267	4.876934e-05	2	2	222.180	2.775382e-05	1	1
222.180	2.223863e-05	2	2	222.180	2.223863e-05	2	2
222.180	2.223863e-05	2	2	222.180	2.775382e-05	1	1
223.869	2.973496e-05	2	2	223.869	3.710925e-05	1	1
223.869	2.973496e-05	2	2	223.869	2.973496e-05	2	2
223.869	3.710925e-05	1	1	223.869	2.099584e-05	0	0
226.042	3.092474e-05	1	1	226.042	2.477943e-05	2	2
226.042	3.092474e-05	1	1	226.042	2.477943e-05	2	2
226.042	2.477943e-05	2	2	457.058	2.008014e-05	1	1
457.058	2.008014e-05	1	1	514.258	5.257589e-05	1	1
514.258	4.212810e-05	2	2	514.258	5.257589e-05	1	1
514.258	2.974663e-05	0	0	514.258	4.212810e-05	2	2
514.258	4.212810e-05	2	2	517.195	4.400219e-05	2	2
517.195	5.491475e-05	1	1	517.195	5.491475e-05	1	1
517.195	3.106993e-05	0	0	517.195	4.400219e-05	2	2
517.195	4.400219e-05	2	2	518.947	3.519574e-05	1	1
518.947	3.519574e-05	1	1	523.999	3.800463e-05	1	1
523.999	3.800463e-05	1	1	523.999	2.150244e-05	0	0
531.863	3.321211e-05	0	2	531.863	2.650049e-05	1	3
531.863	2.650049e-05	1	3	538.241	2.135472e-05	3	5
538.241	2.135472e-05	3	5	538.241	6.907221e-05	0	2
538.241	3.590646e-05	2	4	538.241	2.135472e-05	3	5
538.241	3.590646e-05	2	4	538.241	2.135472e-05	3	5
538.241	3.590646e-05	2	4	538.241	5.511385e-05	1	3
538.241	5.511385e-05	1	3	546.154	3.149666e-05	0	2
546.154	2.513170e-05	1	3	546.154	2.513170e-05	1	3
547.843	2.189239e-05	2	4	547.843	4.211374e-05	0	2
547.843	2.189239e-05	2	4	547.843	2.189239e-05	2	4
547.843	3.360325e-05	1	3	547.843	3.360325e-05	1	3
550.016	3.509520e-05	0	2	550.016	2.800304e-05	1	3
550.016	2.800304e-05	1	3	731.462	5.251151e-05	2	2
731.462	6.553439e-05	1	1	731.462	5.251151e-05	2	2
731.462	6.553439e-05	1	1	731.462	3.447378e-05	3	3
731.462	3.447378e-05	3	3	731.462	3.707835e-05	0	0
731.462	3.447378e-05	3	3	731.462	3.447378e-05	3	3
731.462	5.251151e-05	2	2	731.888	3.292854e-05	1	1
731.888	2.638504e-05	2	2	731.888	2.638504e-05	2	2
731.888	2.638504e-05	2	2	731.888	3.292854e-05	1	1
738.127	3.363761e-05	2	2	738.127	2.208307e-05	3	3
738.127	4.197975e-05	1	1	738.127	3.363761e-05	2	2
738.127	2.208307e-05	3	3	738.127	4.197975e-05	1	1
738.127	2.208307e-05	3	3	738.127	3.363761e-05	2	2
738.127	2.375150e-05	0	0	738.127	2.208307e-05	3	3
741.128	3.255541e-05	1	1	741.128	3.255541e-05	1	1
741.128	2.608605e-05	2	2	741.128	2.608605e-05	2	2
741.128	2.608605e-05	2	2	781.032	2.278812e-05	0	2
781.901	2.205560e-05	0	2	838.232	3.101684e-05	2	4
838.232	3.101684e-05	2	4	838.232	3.101684e-05	2	4
838.232	4.760864e-05	1	3	838.232	4.760864e-05	1	3
838.232	5.966619e-05	0	2	841.169	3.239664e-05	2	4
841.169	3.239664e-05	2	4	841.169	3.239664e-05	2	4
841.169	4.972653e-05	1	3	841.169	4.972653e-05	1	3
841.169	6.232047e-05	0	2	842.921	3.994218e-05	0	2
842.921	3.187052e-05	1	3	842.921	3.187052e-05	1	3
847.973	4.312988e-05	0	2	847.973	3.441404e-05	1	3
847.973	3.441404e-05	1	3	912.752	3.099871e-05	3	3
912.752	4.721818e-05	2	2	912.752	5.892831e-05	1	1
912.752	4.721818e-05	2	2	912.752	3.099871e-05	3	3
912.752	4.721818e-05	2	2	912.752	5.892831e-05	1	1
912.752	3.099871e-05	3	3	912.752	3.099871e-05	3	3
912.752	3.334074e-05	0	0				

III. COMPARISON TD-DFT – SYMMETRY

A comparison of the potential energy curves obtained by TD-DFT and regular DFT is presented in Figure 2.

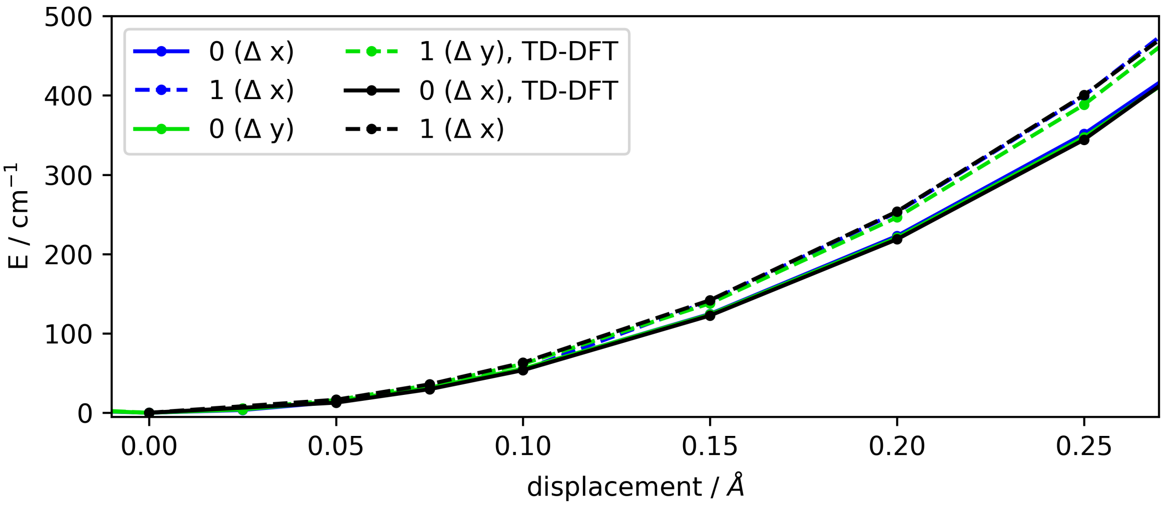

FIG. S2: Potential energy curves for two different electronic states(0/1) while displacing the NO molecule within C_{60} (Δx, Δy, Δz). For both displacements, Δx and Δy, almost perfectly congruent curvatures are obtained.

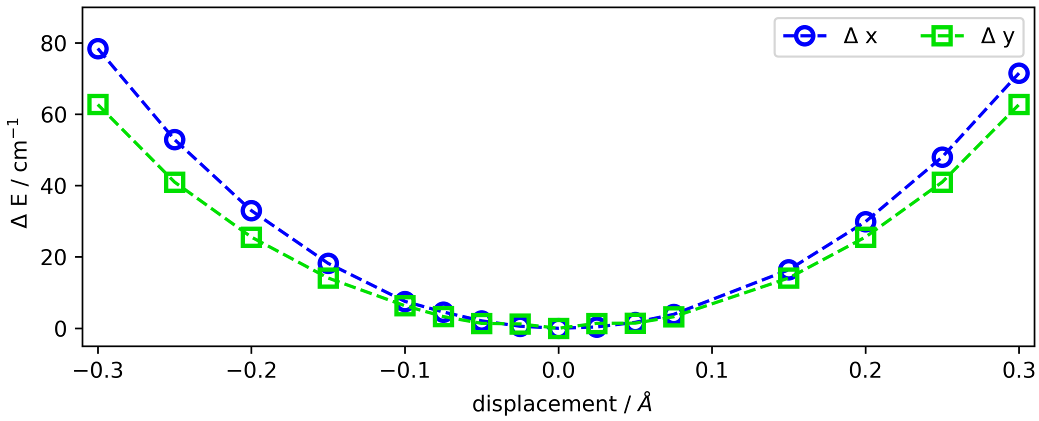

FIG. S3: Energy difference of the two formerly degenerate states, plotted as a function of the NO displacement in x (blue) and y (green) direction.

IV. VIBRATIONAL MODES IN C_{3v}

In this section we compare the vibrational modes of pristine C_{60} to the NO@C_{60} endofullerene with respect to intensity and line position. Note the minimal impact of the guest molecule on the vibrational modes of the cage.

TABLE IV: Vibrational modes below 522 cm^{-1} of C_{60} and NO@C_{60} computed by DFT using the PBE functional and D3 correction.

C_{60}		NO@C_{60}	
E / cm^{-1}	I / $\frac{km}{mol}$	E / cm^{-1}	I / $\frac{km}{mol}$
		122.5366	0.0177
		122.5366	0.0177
		165.6659	0.0130
258.2293	0.0000	259.7283	0.0000
258.2293	0.0000	259.7700	0.0000
258.4928	0.0000	263.2098	0.0009
258.6981	0.0000	263.2150	0.0010
258.6981	0.0000	264.7619	0.0001
335.7735	0.0001	337.3567	0.0005
335.7735	0.0001	341.2216	0.0066
336.1192	0.0038	341.2795	0.0064
346.3185	0.0028	347.9088	0.0000
346.3185	0.0028	351.5452	0.0009
346.5128	0.0041	351.5868	0.0009
346.8019	0.0000	352.5972	0.0007
397.5587	0.0000	398.1568	0.0005
397.5587	0.0000	398.1932	0.0000
397.8295	0.0014	398.4363	0.0000
397.8295	0.0014	398.4372	0.0000
397.8711	0.0000	398.4402	0.0000
424.1653	0.0000	428.1476	0.0011
424.1653	0.0000	428.3596	0.0001
424.1718	0.0001	428.5120	0.0001
424.3658	0.0000	430.3513	0.0000
424.3658	0.0000	430.3534	0.0000
476.9995	0.0000	481.7027	0.0000
477.0475	0.0002	482.0317	0.0003
477.4045	0.0000	482.8297	0.0004
477.4045	0.0000	482.8665	0.0004
491.3467	0.0000	493.3423	0.0007
521.7409	24.2570	522.6215	23.0155
521.8968	24.5445	522.7743	23.4113
521.8968	24.5445	522.7813	23.3916
